# The Assembly of Individual Chaplin Peptides from *Streptomyces coelicolor* into Functional Amyloid Fibrils

**DOI:** 10.1371/journal.pone.0018839

**Published:** 2011-04-19

**Authors:** Elizabeth B. Sawyer, Dennis Claessen, Maria Haas, Bhavna Hurgobin, Sally L. Gras

**Affiliations:** 1 Department of Chemical and Biomolecular Engineering and the Bio21 Molecular Science and Biotechnology Institute, The University of Melbourne, Parkville, Australia; 2 Department of Molecular and Developmental Genetics, Institute of Biology, Leiden University, Leiden, the Netherlands; Massachusetts Institute of Technology, United States of America

## Abstract

The self-association of proteins into amyloid fibrils offers an alternative to the natively folded state of many polypeptides. Although commonly associated with disease, amyloid fibrils represent the natural functional state of some proteins, such as the chaplins from the soil-dwelling bacterium *Streptomyces coelicolor*, which coat the aerial mycelium and spores rendering them hydrophobic. We have undertaken a biophysical characterisation of the five short chaplin peptides ChpD-H to probe the mechanism by which these peptides self-assemble in solution to form fibrils. Each of the five chaplin peptides produced synthetically or isolated from the cell wall is individually surface-active and capable of forming fibrils under a range of solution conditions *in vitro*. These fibrils contain a highly similar cross-β core structure and a secondary structure that resembles fibrils formed *in vivo* on the spore and mycelium surface. They can also restore the growth of aerial hyphae to a chaplin mutant strain. We show that cysteine residues are not required for fibril formation *in vitro* and propose a role for the cysteine residues conserved in four of the five short chaplin peptides.

## Introduction

Most bacteria can form multicellular communities known as biofilms, in which cells are protected from harsh environmental conditions. Recent advances have led to the identification of a number of fibrous proteins that are associated with the extracellular matrices of such microbial communities. These proteins, which closely resemble amyloid fibrils, include the *Escherichia coli* curli proteins [1] and the *Bacillus subtilis* TasA protein [2]. Likewise, amyloid fibrils are present on the cell surface of many filamentous organisms such as those formed by the SC3 hydrophobin protein from the filamentous fungus *Schizophyllum commune* [3], [4] or the chaplins of the filamentous bacterium *Streptomyces coelicolor* [5], [6], [7]. In diseases associated with protein misfolding, such as Alzheimer's disease, the appearance of amyloid fibrils is correlated with disease progression, although it is not clear whether these fibrils contribute directly to toxicity [8], [9]. In contrast, the fibres associated with the extracellular matrix or cell walls in microbial communities are thought to play a functional role, assisting in cell adhesion, the formation of biofilms or flocs and the maintenance of complex multicellular communities. These ‘misfolded’ β-sheet-rich structures represent low-energy conformations that are considered generic to the protein backbone and therefore accessible to all proteins [10], [11], [12]. The study of microbial fibres will further increase our understanding of such protein structures and provide a useful comparison for toxic or disease-related fibrils. Such investigations will also aid the development of novel protein-based materials, as these hydrophobic peptides and self-assembling fibrils are attractive targets for biotechnology [13], [14] with proposed applications as diverse as window coatings, emulsifiers in consumer products such as ice cream [15] and fibrous materials and scaffolds.

In 2003, the chaplins (for ***c*** *oelicolor* **h**ydrophobic **a**erial **p**roteins) were discovered in the common Gram-positive soil bacterium *S. coelicolor* [5], [6]. This group of eight proteins (ChpA-H) coat the aerial hyphae assisting spore dispersal and the colonisation of surrounding soil. That *S. coelicolor* produces eight amyloidogenic proteins is highly unusual as other systems such as those found in *B. subtilis* and *E. coli* have only one major amyloidogenic component, which is expressed alongside accessory proteins that direct or seed the assembly process. The eight chaplin proteins share significant sequence identity [5], [6] (Table 1), including a highly conserved hydrophobic ‘chaplin’ domain approximately 40 amino acids in length. ChpA-C have two N-terminal chaplin domains and a C-terminal sorting signal that targets them for sortase-mediated covalent attachment to the *S. coelicolor* cell wall, while ChpD-H are smaller proteins (5–6 kDa) containing a single chaplin domain following an N-terminal secretion signal peptide [5], [6].

**Table 1:** Sequence identities of the chaplins.

	ChpA	ChpB	ChpD	ChpE	ChpF	ChpG
**ChpB**	41.7					
**ChpC**	46.4	46.3				
**ChpE**			60			
**ChpF**			78.8	56.4		
**ChpG**			69.8	50	68.3	
**ChpH**			80.8	52.8	86.5	69.8

Percentage sequence identities are given for the sequences of mature peptides following cleavage of N-terminal signal peptides.

The current model for chaplin function, based on the differential expression of *chp* genes throughout the cell cycle, suggests that the chaplins adopt distinct roles *in vivo*. Two short chaplins, *chpE* and *chpH*, are expressed at high levels during both the vegetative and aerial mycelial phases, whereas the other *chp* genes are only expressed during aerial hyphae formation. ChpE and ChpH, are thought to serve two functions - firstly acting as surfactants to lower the surface tension of water, then assembling (presumably by heteropolymerisation with the other short and long chaplins) into a hydrophobic layer that coats the emerging hyphae [6], [16]. The expression of *chpE* early in the *S. coelicolor* developmental cycle and the observation that this gene is essential in a wild-type genetic background (intriguingly *chpE* is dispensable in strains lacking other *chp* genes or with null mutations in the Tat secretion system [17]), suggests a unique function for ChpE amongst the chaplins. This peptide, which lacks the two cysteine residues conserved across all other chaplin domains, has orthologues in other *Streptomyces* species and is thought to coordinate the assembly of the other chaplins into the pair-wise aligned rodlet structures that comprise the hydrophobic coat. This process is also mediated by the two rodlin proteins, RdlA and RdlB, that are secreted by growing aerial hyphae [18].

The co-existence of genes for both long and short chaplins in sporulating actinomycetes appears to be universal, although the numbers of these genes may vary, with ChpC, ChpE and ChpH seeming to constitute the smallest group of components required to form a chaplin apparatus [6]. The roles of individual chaplins have been addressed in an *in vivo* model by the construction of a “minimal strain” that expresses only ChpE and has a *bld* colony phenotype (i.e. is unable to form aerial hyphae) [17]. Reintroduction of the three long chaplins, ChpABC, did not improve aerial growth, but expression of *chpH* did enable the formation of sparse aerial mycelium. When *chpC* was expressed alongside *chpH*, the bacteria produced a robust sporulating aerial mycelium, albeit at a reduced rate compared to the wild type; similar results were obtained when *chpAD* were reintroduced. Interestingly when the conserved cysteine residues were removed from *chpH* by site-directed mutagenesis, formation of aerial hyphae in the “minimal strain”+chpC+chpH* was severely compromised and fibril morphology was altered [17]. These results indicate some degree of redundancy among the chaplins, and that expression of both long and short chaplins, with at least one short chaplin containing the conserved cysteine motif, is vital for the development of robust aerial hyphae.

Despite the wealth of genetic and microbiological data available, significant gaps remain in our understanding of chaplin function and assembly at the molecular level. For example, it is not known whether individual chaplin peptides are capable of forming fibrils and whether fibres made from different individual chaplins are morphologically or structurally distinct. Neither has the role of disulphide bonding in fibril formation and chaplin function been described. To investigate the assembly of the short chaplins and to probe the role of disulphide bonding in fibril formation, we have undertaken a biophysical characterisation of individual chaplin peptides synthesised using standard FMOC chemistry or peptides isolated and purified from the cell wall of *S. coelicolor*. We examine the secondary and internal core structure of the fibrils formed by the chaplin peptides, compare the secondary structure of fibrils formed *in vitro* with those formed *in vivo* on the cell wall and test the ability of the chaplins to assemble into fibrils under reducing conditions.

## Results and Discussion

### Bioinformatic analyses reveal subtle differences between the short chaplins

The chaplin proteins share the highly conserved chaplin domain that contains approximately 40 hydrophobic residues; the long chaplins have two of these domains, whilst the short chaplins have just one. A series of bioinformatic comparisons were conducted to identify other similarities and differences between the chaplin sequences, including the propensity of the short chaplin sequences to form β-sheet secondary structure and amyloid fibrils.

**Figure 1:**
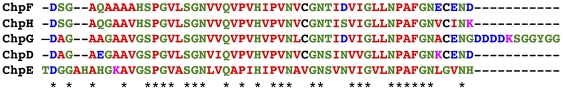
Sequence alignment of the five short chaplin peptides. Sequence alignment of ChpD-H generated using Lalign [19]. Residues conserved in all five peptides are indicated with an asterisk.

The alignment and sequence identities of the mature proteins calculated using Lalign [19] are shown in Figure 1 and Table 1, respectively. The percentage identities are higher amongst the short chaplins than the long chaplins, reflecting the relative number of “non-chaplin domain” residues in these larger proteins. However, sequence identity is high across all eight proteins, with the closest alignment between ChpF and ChpH (86.5%). The identity scores for ChpE are consistently lower due to the absence of the two cysteines that are conserved across all other chaplin proteins and the presence of 4 extra residues in the N-terminal region of ChpE (Figure 1). Most of the other differences in sequence are substitutions between similar residues (S/T or I/V) or occur in the C-terminal region after the second conserved cysteine – this region contains a higher proportion of charged residues than any other stretch of sequence and is largely responsible for the differences in isoelectric point (pI) between the short chaplins.

The pI of ChpD, ChpF and ChpG are between 3.80 and 4.49, while ChpE and ChpH have a pI of 6.82 and 6.90, respectively. This means that at any given pH there will be a significant difference in the net charge of the chaplins, which may have important implications for assembly. For example, at pH 7 under conditions similar to those encountered by *S. coelicolor* in its environment, ChpD, ChpF and ChpG will have charges of −3, −4.5 and −6 respectively whereas ChpE and ChpH will be positively charged (+1 and +0.5 respectively) favouring electrostatic interactions between the two groups of peptides.

The five short chaplins exhibit considerable similarity in terms of their hydrophobicity profiles, as expected given their high sequence identity. The Grand average of hydropathicity (GRAVY) of a polypeptide sequence is the sum of the hydropathy values of all amino acids, divided by the number of residues in the sequence. The chaplins are very hydrophobic with GRAVY scores of between 0.163 (ChpG) and 0.600 (ChpH) with the most hydrophobic region found between residues 15 and 43. The lower score of ChpG can be largely attributed to the presence of an 11 residue C-terminal tail consisting mostly of aspartic acid and glycine residues. In general, the C-termini of ChpD, ChpF and ChpG are slightly less hydrophobic than those of ChpE and ChpH.

Secondary structure predictions for the five short chaplins obtained using Jpred [20], PredictProtein [21] and TANGO [22], [23], [24] are in good agreement. The chaplins have a very low propensity to form α-helical structure, the only exception being 9 residues near the N-terminus of ChpF and all five proteins have a relatively high propensity to form β-turn and β-sheet structures. The presence of conserved proline residues at normalised residue positions 15, 26, 30 and 46 means that any structural predictions tend to be short-range, as proline is a known breaker of secondary structural elements. Proline is commonly found at the edges of β-sheets and in β-turns, which may account for the high β-turn propensities predicted for the chaplins.

An increasing number of intrinsically unstructured proteins (proteins characterised by a lack of stable tertiary structure) have been described in recent years. In many cases these proteins adopt a fixed three-dimensional structure only after binding to other proteins or ligands. Given the short ranges of the secondary structures predicted for the chaplins and their proposed function to self-assemble into fibrous structures, the algorithm VLXT within PONDR [25], [26], [27] was used to assess the propensity of the chaplin sequences towards disorder. PONDR uses information about local amino acid composition, flexibility and hydropathy to predict all non-rigid regions including random coils, partially unstructured regions and molten globules.

It was found that the chaplins cluster into two groups: ChpD, ChpF and ChpG were predicted to be largely disordered, with a short region of order between residues 30–45; ChpE and ChpH were predicted to have disordered N-terminal regions but some structure within the ∼20 C-terminal residues. The similar grouping of the chaplins with respect to pI, GRAVY scores and propensity for disorder may have some significance as ChpE and ChpH are thought to have a different role, assembling into a surfactant film at the air-water interface at the onset of aerial hyphae development before formation of the rodlet layer [5], [6].

The algorithm TANGO [22], [23], [24] was used to predict the propensity of the chaplins to form cross-β aggregates. TANGO predicted that the chaplins all have a high propensity towards cross-β aggregation. This is consistent with our experimental observations, described below. The most aggregation-prone region was predicted to be between normalised residue positions 36–46, suggesting that these residues may drive aggregation and β-sheet assembly.

### Properties of the chaplin peptides in solution

The chaplin proteins are remarkable in their ability to assemble rapidly on agitation. To probe changes in the secondary structure of the individual short chaplins, CD spectra were recorded for each of the synthetic chaplin peptides before and after vortexing; conditions previously shown to induce a random coil to β-sheet transition for a solution of mixed chaplin peptides extracted from *S. coelicolor* cell walls [5].

**Figure 2:**
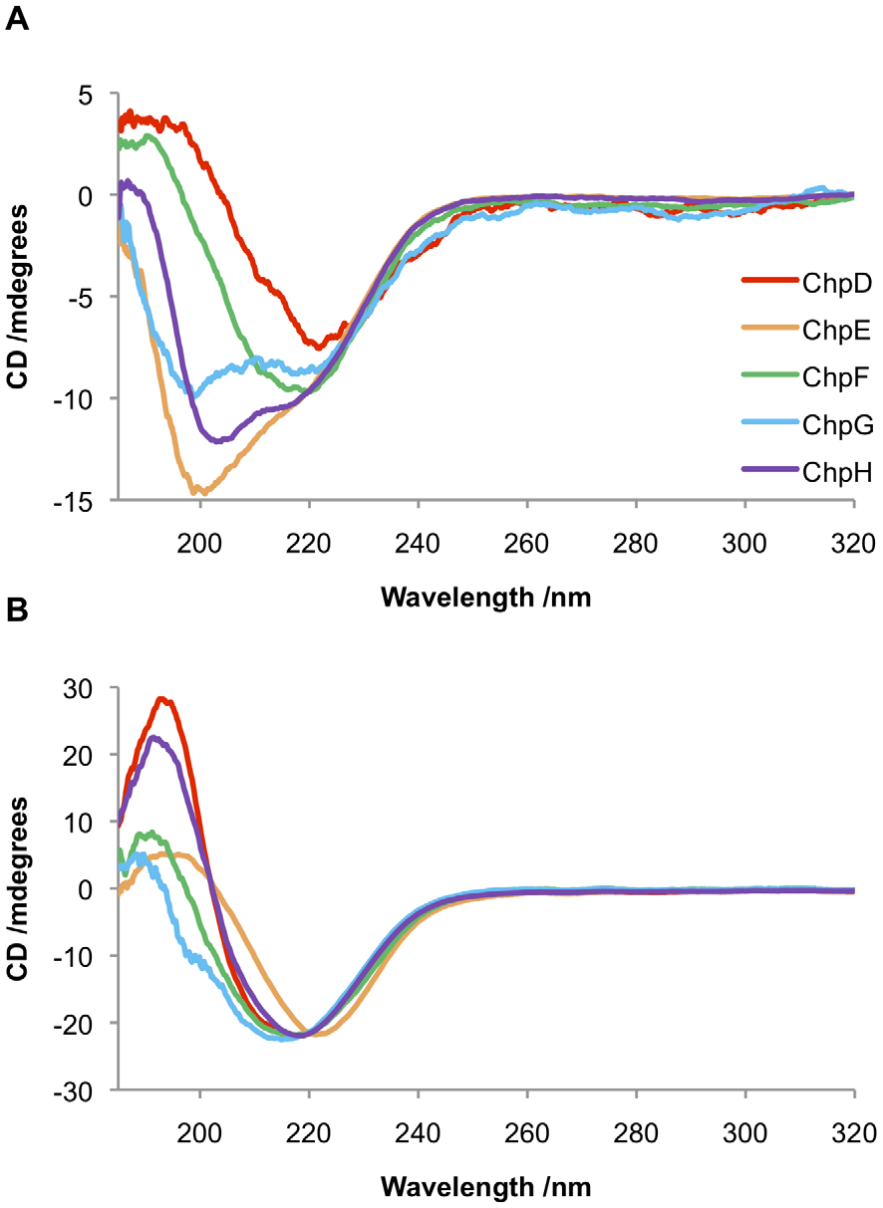
Characterisation of the secondary structures of chaplin peptides by CD spectroscopy. (A) Peptides in water without agitation. ChpD (red) and ChpF (green) are comprised largely of β-sheet; ChpE (orange) is predominantly random coil; while the spectra of ChpG (blue) and ChpH (purple) are indicative of a mixed secondary structure comprising elements of both β-sheet and random coil. (B) Upon vortexing all of the chaplins adopt a β-sheet rich secondary structure.

The CD spectra of peptides prior to vortexing (Figure 2A) reveal subtle differences in secondary structure between the chaplins: ChpD and ChpF appear to comprise β-sheet; ChpE is predominantly random coil; while the spectra of ChpG and ChpH are indicative of a mixed secondary structure comprising elements of both β-sheet and random coil. Although the peptides were expected to be predominantly monomeric under these conditions, given the high propensity of the chaplin peptides to aggregate it is possible that the β-sheet structures observed by CD could be due to partial aggregation. An alternate explanation is that the β-sheet structures observed could represent genuine secondary structural elements, consistent with the predictions made by Jpred, PredictProtein and TANGO. Upon vortexing all samples adopt a β-sheet-rich secondary structure, as shown in Figure 2B.

The chaplins also act as surfactants and a solution of mixed proteins extracted from the cell wall of *S. coelicolor* has previously been reported to lower the surface tension of water from 72 mJ.m^−2^ to 26 mJ.m^−2^ [5]. This process is thought to assist aerial hyphae as they protrude from a body of water prior to the dispersal of spores. The surfactant properties of each of the chaplin peptides was tested using a pendant droplet method similar to that described by Claessen et al. [5].

At low concentrations (∼100 µg.ml^−1^) and under conditions where the chaplin peptides are expected to be monomeric each of the peptides behaved similarly, lowering the surface tension by 16–27 mJ.m^−2^ within seconds and then further towards a final surface tension of ∼30–40 mJ.m^−2^ over 10 minutes (Table 2). The differences in surface tension between individual chaplins appear small and are not of sufficient magnitude to indicate any individual roles for the peptides. Interestingly at high concentrations (>500 µg.ml^−1^) or when aggregation is induced the ability of the chaplins to act as surfactants is reduced (Table 2). This finding suggests that chaplin peptides released into solution may alter the surface tension, rather than fibrils formed on the surface of aerial hyphae, consistent with a model where the chaplin peptides act firstly as surfactants to lower the surface tension of water and secondly to assemble into a hydrophobic layer that coats the hyphae [6], [16].

**Table 2:** Interfacial tension measurements for solutions of chaplins after treatment with trifluoroacetic acid.

	Interfacial tension/mJ.m^−2^							
	100 µg.ml^−1^				>500 µg.ml^−1^			
	t = 0 minutes		t = 10 minutes		Before vortexing		After vortexing	
**ChpD**	52	±5	34	±4	72	±1	72	±1
**ChpE**	53	±2	32	±5	68	±3	71	±1
**ChpF**	45	±5	28	±3	66	±4	69	±2
**ChpG**	56	±3	37	±1	72	±1	72	±1
**ChpH**	51	±4	42	±1	65	±2	69	±3

At low concentrations (∼100 µg.ml^−1^) the chaplins lower the surface tension of water considerably within 10 minutes; at concentrations >500 µg.ml^−1^ the chaplins no longer act as surfactants either before or after vortexing. Water has an IFT of 72.80 mJ.m^−2^.

### Synthetic peptides ‘rescue’ chaplin-deficient strains of *S. coelicolor*

**Figure 3:**
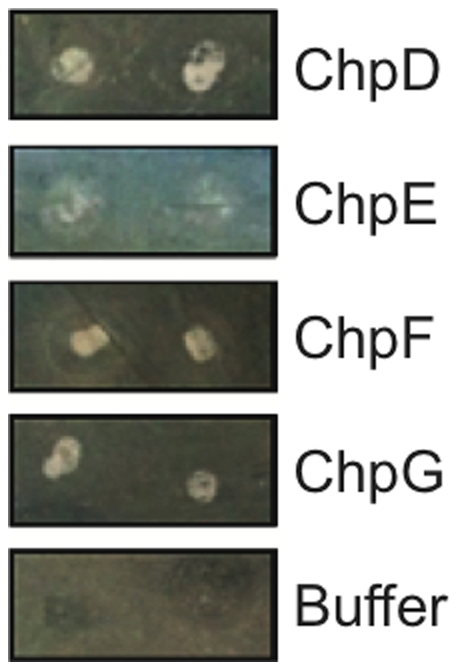
Extracellular complementation of *ΔchpABCDEH* colonies with synthetic chaplin peptides. Each peptide was able to induce the formation of aerial hyphae, indicated by the appearance of a white, fluffy layer in the area where the chaplin peptide was applied to the colony.

The ability of synthetic chaplin peptides to ‘rescue’ a chaplin-deficient strain of *S. coelicolor* was assessed by extracellular complementation. As shown in Figure 3, application of ChpD, ChpF or ChpH to *ΔchpABCDEH* colonies restored the formation of aerial hyphae within 16 hours; ChpE was also able to complement *ΔchpABCDEH* cells within the same timescale but the formation of aerial hyphae was less robust than when ChpD, ChpF or ChpH were applied to the colony surface. In contrast, colonies treated with buffer only produced no aerial hyphae within this timeframe. The fact that ChpE is least efficient in restoring growth to *ΔchpABCDEH* colonies supports the hypothesis that ChpE has a specialised, perhaps coordinating role in the chaplin polymerisation process [17]. We hypothesise that the ability of peptides applied extracellularly to restore development is due to their effect in lowering the surface tension of the medium enabling hyphae to extend into the air.

### Each of the short chaplin peptides forms fibrillar aggregates *in vitro*

**Figure 4:**
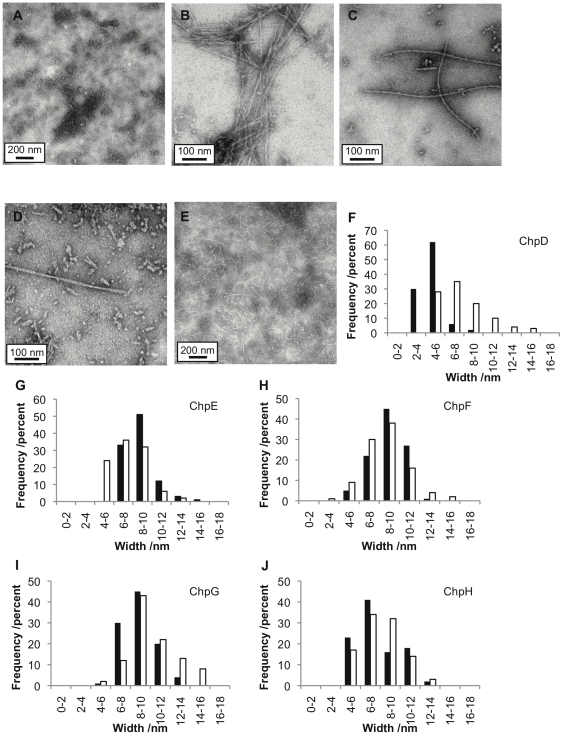
Transmission Electron Microscopy analysis of chaplin fibrils. TEM images of chaplin fibrils formed at pH 7: (A) ChpD, scale bar 200 nm; (B) ChpE, scale bar 100 nm; (C) ChpF, scale bar 100 nm; (D) ChpG, scale bar 100 nm; (E) ChpH, scale bar 200 nm. Histograms showing width distributions of chaplin fibrils at pH 2 (closed bars) and pH 7 (open bars) (F) ChpD; (G) ChpE; (H) ChpF; (I) ChpG; (J) ChpH. The sample size was *n*>100 for all measurements.

The formation of ChpD, ChpE, ChpF, ChpG, and ChpH fibrils in vortexed solutions of individual synthetic chaplin peptides was confirmed by Transmission Electron Microscopy (TEM). Fibrils formed readily from each peptide under a variety of solution pH and closely resembled those formed from solutions of individual peptides extracted from the cell wall and isolated by High Performance Liquid Chromatography (HPLC). As shown in Figure 4, chaplin fibrils vary considerably in width and length both within and between different samples, although the width distributions and modal widths observed are similar for each chaplin. At all concentrations tested (0.1–2 mg.ml^−1^) chaplin fibrils exhibited a strong tendency to associate and clump together.

The uniformity in width distribution of synthetic chaplin fibrils was largely unaffected by sample age or pH. Fibrils examined after 72 hours or four weeks exhibited similar width distributions. In general, chaplin fibrils range of 3.8 nm–17.6 nm in width with a mode of 6 nm–10 nm. The observation of a broad range of fibril widths may be due to the presence of fibrils at different stages of maturity, from protofilaments to mature fibrils. Interestingly, ChpD fibrils formed at pH 2 were much narrower than those in the other samples, with widths ranging from 2.2 nm–8.3 nm with a mode of 4 nm–6 nm. ChpH fibrils generally had a smaller range of widths (4.2 nm–13.5 nm) than observed for other chaplin fibrils regardless of pH, suggesting either that these fibrils cannot mature beyond the protofilament stage (at least within the time frame of four weeks) or that the mature fibril consists of fewer strands than the fibrils of other chaplins.

The fibrils formed by the individual synthetic peptides or chaplin peptides purified from the cell wall also resemble fibrils formed from a mixture of synthetic peptides or a mixture of peptides present in the crude cell extract. This suggests that the combination of the five chaplin peptides does not inhibit or dramatically alter amyloid fibril assembly. It is not yet clear whether the fibrils formed in these solutions consist of multiple different chaplins or whether fibrils form from individual chaplins in the mixture.

### Chaplin fibrils exhibit cross-β X-ray diffraction patterns

**Figure 5:**
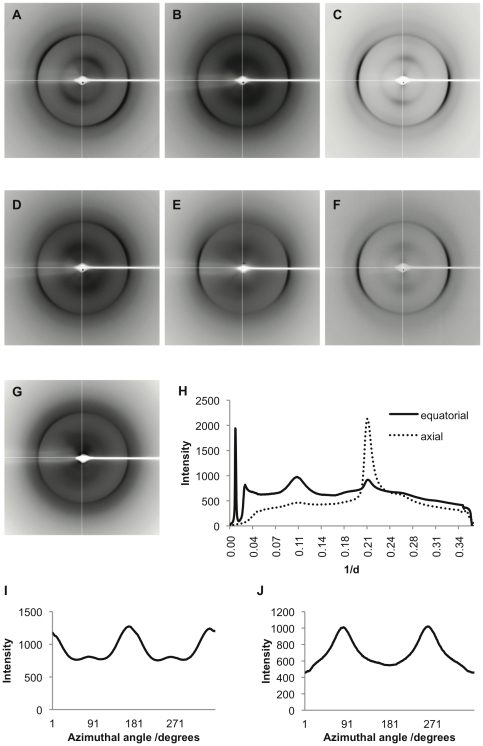
X-ray fibre diffraction reveals chaplin fibrils have a cross-β core structure. X-ray fibre diffraction patterns of ChpD (A); ChpE (B); ChpF (C); ChpG (D); ChpH (E); fibrils formed from a mixed solution of synthetic peptides (F); and crude cell wall extract (G). Each stalk gave rise to anisotropic axial inter-strand reflections at ∼4.7 Å and equatorial inter-sheet reflections at ∼10 Å typical of a cross-β core structure. Diffraction patterns were analysed by radial integration along both axial (dotted line) and equatorial (solid line) axes (H). The variation in X-ray intensity with azimuthal angle is shown for the 4.7 Å (I) and 10 Å (J) reflections in the ChpE fibril sample in image F.

Analysis of the substructures of fibrils made from individual synthetic peptides, mixed synthetic peptides or from the crude cell wall extract by X-ray fibre diffraction confirmed that these fibrils have highly similar core structures. Stalks from each sample gave rise to anisotropic X-ray diffraction patterns displaying the typical features of a cross-β substructure, with axial inter-strand reflections at ∼4.7 Å and equatorial inter-sheet reflections at ∼10 Å (Figure 5A–G). The positions of these reflections are given in Table 3 and a typical radial intensity profile in the axial and equatorial direction provided in Figure 5H. The variation in X-ray intensity as a function of azimuthal angle is also shown for the reflections at 4.7 Å and 10 Å in the ChpE diffraction pattern further illustrating the perpendicular orientation of these reflections (Figure 5I–J).

**Table 3:** Positions of the inter-strand and inter-sheet reflections obtained from X-ray diffraction of chaplin fibrils.

	Position of Reflection/Å			
**ChpD**	4.72	±0.01	10.23	±0.65
**ChpE**	4.73	±0.01	9.54	±0.05
**ChpF**	4.69	±0.02	9.99	±0.29
**ChpG**	4.77	±0.02	10.10	±1.09
**ChpH**	4.73	±0.03	9.97	±0.74
**Mixed synthetic**	4.73	±0.03	9.71	±0.14
**Crude extract**	4.77	±0.02	10.99	±1.97

Fibrils made from individual chaplin peptides, solutions containing a mixture of synthetic peptides or the crude cell wall extract all gave rise to axial inter-strand reflections at ∼4.7 Å and equatorial inter-sheet reflections at ∼10 Å indicative of a cross-β fibril structure.

These data provide the first conclusive evidence that the fibrillar aggregates formed by the chaplins are composed of stacked β-sheets and thus satisfy the cross-β definition of amyloid fibrils. Moreover, the positions of these reflections confirm that fibrils formed in solutions containing a single peptide or a mixture of synthetic or naturally extracted peptides have the same core structure.

### Chaplin fibrils formed *in vitro* are structurally similar to those formed *in vivo*

Previous work by Claessen et al. suggests that rodlins and chaplins are both required for the formation of the paired rodlet ultrastructure visible on the surface of the spores and aerial mycelium [18], however, the contribution made by each to the formation of these structures is unknown. Fourier transform infra-red (FTIR) microscopy was employed to compare the structures of the outer surface of spores and aerial hyphae produced by the *S. coelicolor* wild-type (strain M145) with the surfaces of a *ΔchpABCDEFGH* mutant that expresses rodlins but no chaplins and a Δ*rdlAB* mutant that expresses chaplins but lacks the rodlin proteins. This method enables a comparison between the secondary structure of chaplin fibrils formed *in vitro* and those assembled on the surface of spores or aerial mycelium of chaplin-expressing strains *in vivo*.

**Figure 6:**
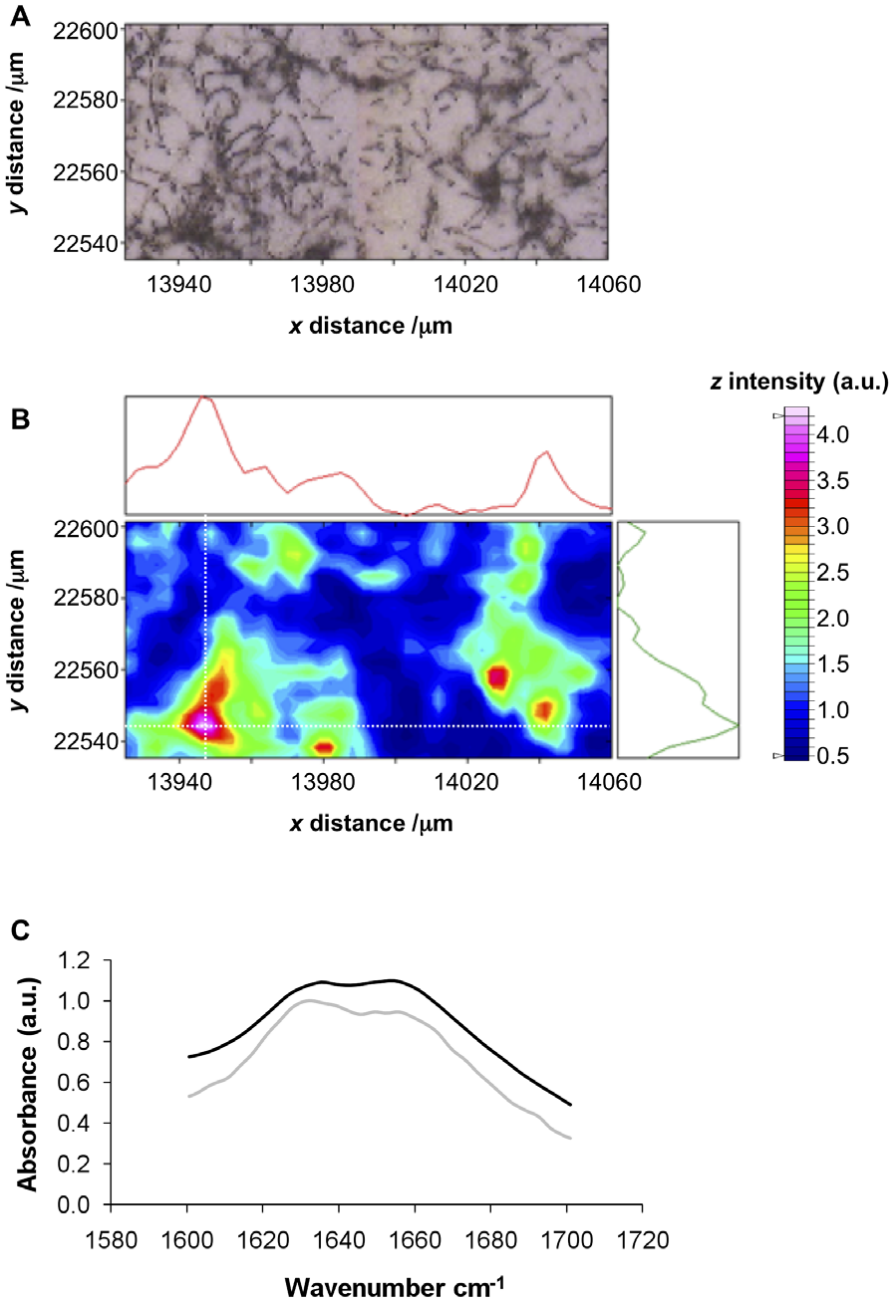
FTIR microscopy of *S. coelicolor* spores and aerial mycelium. (A) Light microscopy image of aerial hyphae and spores of the *S. coelicolor* M145 strain. (B) FTIR intensity in the amide I region for the same region; the coloured bar indicates high to low intensity (pink-blue respectively) and adjoining graphs represent the intensity of absorbance in the amide I region (1600 cm^−1^–1700 cm^−1^) in x- and y- dimensions in the position of the cross-hairs (white dotted lines). (C) FTIR spectra for a region of high amide I intensity indicated by the cross-hairs in the FTIR maps for the M145 strain (light grey) and Δ*rdlAB* strain (black).

The FTIR microscopy map for aerial hyphae and spores of *S. coelicolor* M145 is shown in Figure 6A–B. Regions of high amide I absorbance (1600–1700 cm^−1^) were found to localise with large clusters of spores and aerial hyphae on the surface of the window. Similarly, the Δ*rdlAB* strain that makes chaplins but no rodlins absorbed strongly in the amide I region. The regions of high intensity co-localised with high densities of hyphae and spores (Figure S1). In contrast, the hyphae of the *ΔchpABCDEFGH* mutant did not significantly absorb in the amide I region (Figure S2).

The spectra observed for clumps of wild type and Δ*rdlAB S. coelicolor* spores contained two broad peaks at approximately 1633–1635 cm^−1^ and at 1655 cm^−1^ as shown in Figure 6C. These peaks are indicative of β-sheet secondary structure and either disordered or α-helical content respectively [28]. The similarity of the spectra for the M145 and Δ*rdlAB* strains indicates that chaplin proteins on the surface of the spores contribute to the observed amide I intensity both in the presence and absence of the rodlins. The absence of these peaks in the *ΔchpABCDEFGH* mutant indicates a lack of β-sheet secondary structure on these hyphae. These observations are consistent with a model in which the chaplins are responsible for the formation of β-sheet-rich secondary structures on the surface of spores and hyphae.

The FTIR spectra of chaplin fibrils formed *in vivo* are consistent with the spectra of those assembled *in vitro*. In each case broad peaks in the amide I region were detected at 1630–1635 cm^−1^ and at 1655–1666 cm^−1^ as shown in Table 4. This indicates the structural similarity of fibrils formed *in vitro* with those formed *in vivo*. The formation of chaplin fibrils *in vivo* in the absence of the rodlins also provides evidence that the rodlins do not modify the structure of the chaplin fibrils, although they may alter the arrangement of these fibrils on the spore surface, consistent with previous data [18].

**Table 4:** Positions of peaks observed in FTIR spectroscopy of chaplin fibrils.

Source	Sample	Peak Positions/cm^−1^	
**Synthetic**	**ChpD**	1633	1660 shoulder
	**ChpE**	1632	1660 shoulder
	**ChpF**	1634	1660 shoulder
	**ChpG**	1631	1666
	**ChpH**	1630	1665
**Natural**	**Crude extract**	1630	1673
***In vivo***	**wild-type**	1633–1635	1655
	***ΔrdlAB***	1633–1635	1655

Position of the peak of maximal intensity in the amide I FTIR spectra of fibrils composed of synthetic chaplin peptides, the crude cell wall extract or observed on the cell wall of *S. coelicolor*. Broad peaks were detected at 1630–1635 cm^−1^ and at 1655–1666 cm^−1^, indicative of a β-sheet secondary structure in each of these samples.

### Reduced chaplins form amyloid fibrils that closely resemble those formed under non-reducing conditions

The conserved cysteine residues in ChpD, ChpF, ChpG and ChpH peptides are important features within these short chaplin sequences and in ChpH are known to be essential for the formation of aerial hyphae and assembly of the rodlet layer [17]. Since ChpE lacks these cysteines and yet readily forms amyloid fibrils that are morphologically indistinct from those formed by the other chaplins *in vitro* (Figure 3), we reasoned that disulphide bonding is not a prerequisite for the formation of chaplin fibrils *in vitro*. To test this hypothesis we probed the ability of reduced ChpD, ChpF, ChpG and ChpH to form fibrils.

**Figure 7:**
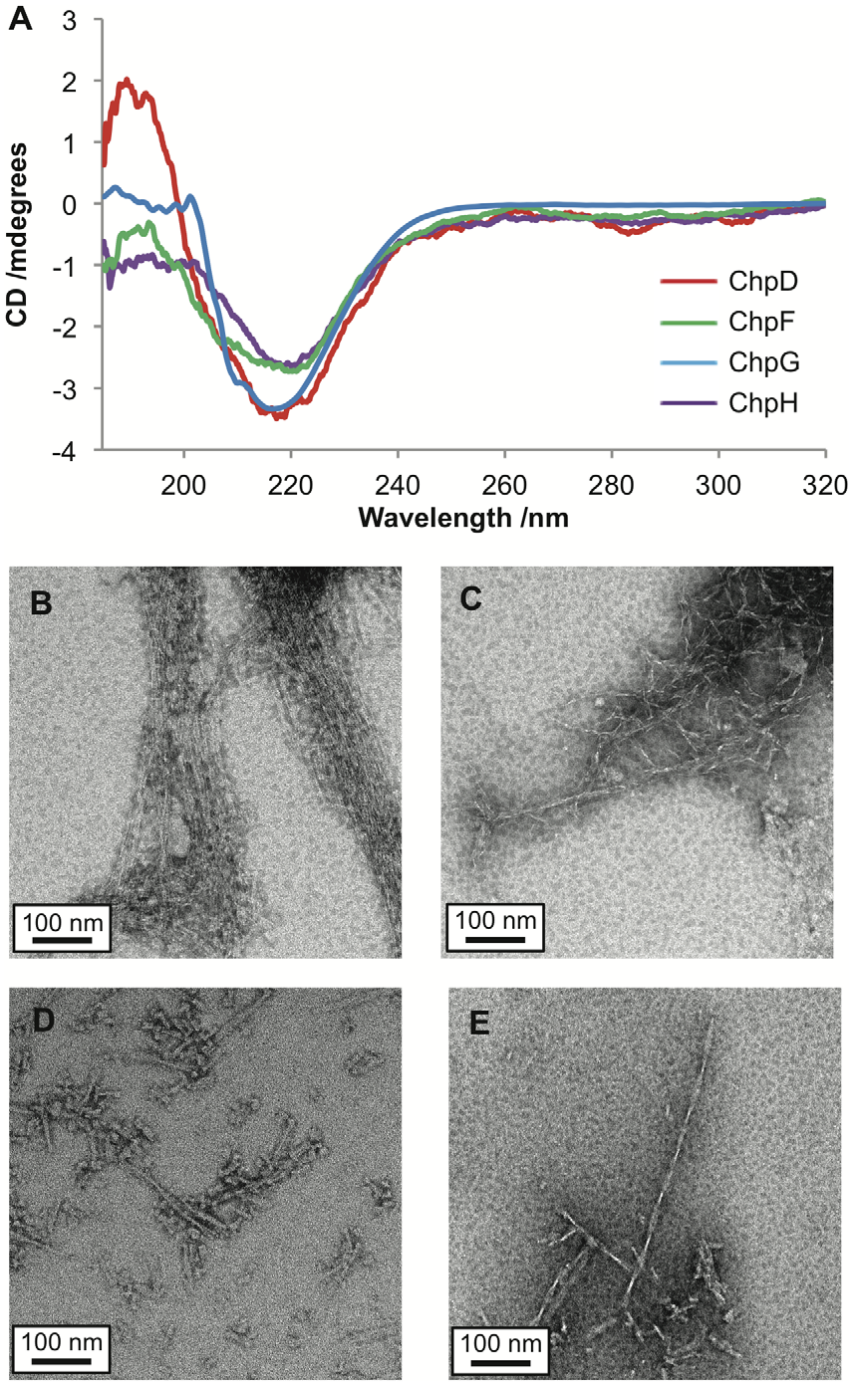
Characterisation of chaplins under reducing conditions. (A) CD spectra of reduced chaplin peptides before vortexing; ChpD (red), ChpF (green), ChpG (blue) and ChpH (purple) secondary structures are predominantly β-sheet. TEM image of fibrils formed from ChpD (D), ChpF (E), ChpG (F) and ChpH (E) under reducing conditions; all scale bars are 100 nm in length.

Peptides were incubated in 1 M dithiothreitol (DTT) in the presence of 8 M urea, then desalted and transferred into a buffer containing 1 mM TCEP. Reduction was confirmed by Mass Spectrometry (data not shown). The CD spectra of reduced ChpD, ChpF, ChpG and ChpH peptides are indicative of a mixed random-coil/β-sheet structure (Figure 7A). Upon vortexing the reduced peptides form fibrillar aggregates. TEM images of fibrils formed from reduced chaplin peptides are given in Figure 7B–E. These fibrils had a similar appearance to those formed under non-reducing conditions – fibril lengths were highly variable and the width distribution was between 3.6 nm and 14.0 nm.

The observation of fibrils formed from chaplin peptides under reducing conditions demonstrates that disulphide bonding is not required for fibril formation. This leads us to question why the bacterial strain expressing only ChpC, ChpE and the Cys-less mutant ChpH* was unable to raise aerial hyphae [17]. One hypothesis is that disulphide bonding between chaplins ensures the formation of stable oligomers that act as critical nuclei in seeding amyloid fibril formation. This hypothesis is supported by the observation of ChpF and ChpH homodimers in preparations of chaplins extracted from the cell wall (Figure S3; extraction method described in Text S1).

A solution of chaplins and other proteins extracted from the cell wall of *S. coelicolor* rapidly forms fibrils upon increased exposure to the air-water interface induced by vortexing. The kinetics of this process were followed using a Thioflavin T (ThT) fluorescence assay. Aggregation proceeds rapidly with no discernable lag phase and in a concentration-dependent manner (Figure S4; ThT assay method described in Text S1). The apparent lack of a lag phase suggests either the presence of seeds or that aggregation proceeds along a downhill energy landscape, a phenomenon that has previously reported for a number of other aggregating peptides in which the critical nuclei can be as small as dimers [29]. Since *Streptomyces* growing in the soil relies on diffusion to expose secreted chaplins to the air-water interface where fibril assembly occurs most rapidly, it is possible that they partially overcome the time limitations of this process by ensuring the formation of amyloidogenic nuclei via disulphide bonding. This would explain why the ChpH* mutant was unable to raise aerial hyphae *in vivo*, whilst accounting for the fact that disulphide bonding is not required for fibril assembly *in vitro*. We hypothesise that the lack of cysteine residues in ChpE, which is expressed early in the developmental cycle like ChpH, favours population of the monomeric, surface-active form of this peptide until formation of the fibrillar cell coat.

In conclusion, we have demonstrated that each of the five short chaplin peptides is capable of forming amyloid fibrils under a range of solution conditions, including reducing conditions. These fibrils have similar morphology and a highly similar cross-β core structure when constructed *in vitro* from single peptides, mixtures of synthetic peptides or naturally extracted peptides. Using FTIR and XRD we have demonstrated that the structures formed by the chaplins *in vitro* are representative of those formed *in vivo* both in the presence and absence of the rodlins. The cysteine residues that are conserved amongst ChpD, ChpF, ChpG and ChpH are not required for fibril formation *in vitro* and we propose a role for these residues in promoting rapid assembly of fibrils by ensuring the formation of critical nuclei by intermolecular disulphide bonding.

Biophysical characterisation of the chaplin peptides represents a significant development in the understanding of functional amyloid fibrils and provides a useful comparison of these structures with fibres associated with diseases, such as Alzheimer's. Our investigations are also of importance in the development of peptide-based materials for bio-/nanotechnology, in particular in applications where tuneable alterations in surface chemistry are required, such as biosensor coatings, because of the surfactant properties of the chaplin peptides.

## Materials and Methods

All materials and reagents were of analytical grade and obtained from Sigma-Aldrich (Castle Hill, NSW, Australia) unless otherwise specified. Chaplin peptides were synthesised according to the published sequences of the mature (N-terminal cleaved) peptides [5] (UniProt accession numbers: ChpD (Q9L1J9); ChpE (Q9X9Z2); ChpF (Q9KYG7); ChpG (Q9KYH3); ChpH (Q9AD92)) in house using a CEM microwave peptide synthesiser (CEM Corporation, NC, USA) or by CS Bio Co. (Menlo Park, CA, USA). Peptides were purified by HPLC to >95% purity and peptide mass confirmed by Mass Spectrometry (ChpD 5070.6; ChpE 5273.9; ChpF 5181.7; ChpG 5993.5; ChpH 5120.8). All buffers were filtered using a 0.2 mm syringe filter (Millipore, Billerica, MA, USA) before use.

### Bioinformatics

Sequence identities were calculated using Lalign [19] with the default scoring matrix, an open gap penalty of −14 and an extend gap penalty of −4. Input sequences were those of the mature peptides following cleavage of the N-terminal signal sequences, using the cleavage position determined by Claessen et al. [5].

The ChpDEFGH sequences were scored for hydrophobicity using the Eisenberg scale [30] and ProtScale [31], via the ExPASy Proteomics Server. Sequences were aligned and the analysis was carried out using the fourth residue of ChpE as a start position. This normalises the window positions to highlight patterns of hydrophobicity between the chaplins. The additional 11 residues at the C-terminus of ChpG do not alter this alignment.

Secondary structure predictions were obtained using Jpred [20], PredictProtein [21] and TANGO [22], [23], [24]. The TANGO algorithm was also used to predict the propensity of the chaplins to form cross-β aggregates at 298 K, pH 7.0 and an ionic strength of 0.225 M (chosen to mimic the ionic strength of a 100 mM solution of phosphate buffer at pH 7.0 with no NaCl adjustment). Predictions of disorder were obtained using PONDR [25], [26], [27].

### Circular Dichroism Spectroscopy

Synthetic chaplin peptides were dissolved at a final concentration of 0.5 mg.ml^−1^ in water and the pH adjusted by titration of NaOH/HCl. For the experiments performed under reducing conditions, peptides were dissolved at a concentration of 0.5 mg.ml^−1^ in a buffer containing 1 M dithiothreitol (DTT), 8 M urea, 0.01% EDTA and 1 M Tris-HCl, pH 8.0. Reduction was then confirmed by Mass Spectrometry and peptides were exchanged into 10 mM phosphate buffer containing 1 mM Tris(2-carboxyethyl)phosphine (TCEP), pH 7.0 using Bio-Spin columns (Bio-Rad, Gladesville, NSW, Australia). Further rounds of MS confirmed that peptides were maintained in a reduced form in the TCEP solution. CD spectra were recorded at 25°C on a JASCO J-815 CD spectropolarimeter (JASCO, Easton, MD, USA) using a 1 mm pathlength cuvette and a step of 0.1 nm, a scan rate of 50 nm.s^−1^ and a scan average of four. Spectra were recorded between 185 nm–320 nm. The acquired spectra were corrected for minor solvent contributions by subtracting the reference spectrum of the buffer.

### Interfacial Tension

Interfacial tension (IFT) measurements were performed using an FTA200 instrument with DropShape Analysis software (version 2.0) (both First Ten Angstoms, Inc., Portsmouth, VA, USA). The instrument was calibrated prior to and at regular intervals throughout data collection using the known IFT of 72.80 mN.m^−1^ for water. Synthetic chaplin peptides were dissolved in trifluoroacetic acid to disrupt any interactions, the acid was then removed by drying under a gentle stream of nitrogen and the peptides were redissolved in 50 mM Tris HCl, pH 6.8 at a final concentration of 100 µg.ml^−1^ (or 500 µg.ml^−1^ for the analysis of aggregated chaplins). A range of droplet sizes between 6.3 µl and 14.9 µl were analysed. Solutions of TFA-treated Tris buffer were also measured as a control – neither TFA treatment nor the presence of 50 mM Tris HCl had any significant effect on IFT.

### Transmission Electron Microscopy (TEM)

Samples were prepared for TEM by adding 6 µl of a 0.5 mg.ml^−1^ solution of fibrils to glow discharged, Formvar and carbon-coated 300 mesh copper TEM grids (ProSciTech, Thuringowa, QLD, Australia). The grids were then washed twice by dipping in water and negatively stained with phosphotungstic acid (ProSciTech) [2% (w/v) solution adjusted to pH 7 with NaOH]; grids were dried with filter paper between each step.

Samples were imaged using a Tecnai G^2^ TF30 microscope (FEI Company, Eindhoven, The Netherlands) operating at 200 kV equipped with a 2 k×2 k CCD camera (Gatan, Pleasanton, CA, USA). Fibril width measurements were performed using ImageJ software (available at http://rsb.info.nih.gov/ij; developed by Wayne Rasband, National Institutes of Health, Bethesda, MD, USA) with *n*≥100 for each sample.

### X-ray Fibre Diffraction

Dried stalk samples were prepared for X-ray fibre diffraction by vortexing a 40 mg.ml^−1^ solution of chaplin peptides (either synthetic or extracted from the cell wall) for 30 minutes at room temperature. Samples were left overnight and then droplets of the fibril-containing solutions were suspended between the ends of two wax-filled capillaries and allowed to dry in air at room temperature as described previously [32]; small stalks of fibrils attached at one end to a capillary were obtained.

X-ray diffraction images of dried fibril stalks were collected on the Macromolecular Crystallography beamline (MX1) at the Australian Synchrotron, Victoria, Australia (wavelength 0.95363 Å) using a Blu-Ice control system [33]. The sample-to-detector distance was 300 mm, with an exposure time of 30 s. Diffraction patterns were converted to tiff files using the program fit-2d (Hammersley/ESRF) and radially integrated to generate one-dimensional scattering patterns using Matlab code, as described previously [34], [35]. The X-ray scattering patterns for dried fibrils were radially integrated in either the equatorial or meridional direction using a 30 degree sector in the azimuthal direction on either side of the reflection. The position of maximal intensity, d, was used to determine the position of reflections and the difference in position of d either side of the pattern used to estimate the error in the position of the reflection. The variation in X-ray intensity for the reflections at 4.7 Å and 10 Å was also determined using the same Matlab code. A calibrant of high density polyethylene (HDPE) was used to determine the position of the beam centre, sample-to-detector distance and pixel size.

### Fourier Transform Infra-red Microscopy/Spectroscopy

*S. coelicolor* strains M145 and Δ*rdlAB* were confluently grown on MS agar at 30°C for 7 days [36]. Sporulating mycelium of *S. coelicolor* was transferred from the agar plates by imprinting onto infrared transparent calcium fluoride windows (22 mm in diameter and 0.5 mm in thickness). The window was gently placed on top of the colony and light pressure applied. Samples were kept dry at room temperature and examined by Fourier Transform Infra Red (FTIR) microscopy within a few hours.

FTIR microscopy experiments were carried out on the Infrared (IR) microscopy beamline at the Australian Synchrotron, Victoria, Australia. Spectra were acquired using a Bruker Hyperion 2000 microscope (Brucker Optics GmbH., Ettlingen, Germany) equipped with a Mercury Cadmium Telluride detector and a sample chamber continuously purged with nitrogen. Mapping experiments were recorded with a tile size of 3 µm and an aperture of 5 µm. Each spectrum was obtained in transmission mode by averaging 128 interferograms. A position on the slide lacking colonies was used for background subtraction using an average of 128 interferograms. Spectral maps were processed in OPUS-NT software 6.5 (Bruker Optics) and data for FTIR maps is presented for 1600–1700 cm^−1^.

FTIR spectra for all other samples were acquired using a Spectrum One FTIR instrument (Perkin Elmer, Waltham, MA, USA). A 1 µl sample of chaplins at 0.5 mg.ml^−1^ was applied to the crystal and allowed to dry forming a thin hydrated film. FTIR was performed in attenuated total reflectance mode and data were collected between 4000–650 cm^−1^ using a scan time of 1 minute and a resolution of 4.0 cm^−1^. Data presented here represent an average of either 4 or 128 interferograms; there was no difference in data averaged from 4 interferograms compared with data averaged from 128 interferograms.

### Complementation Assay

At least 10^7^ spores of the *ΔchpABCDEH* strain [5] were plated on a minimal medium agar plate containing mannitol as the carbon source [36]. After 24 hours of growth at 30°C, 10 µl drops of purified chaplins (50 µg.ml^−1^) or buffer (negative control) were applied in discrete areas on the colony surface, after which plates were incubated for 16 hours. Formation of aerial hyphae was assessed macroscopically, as indicated by the appearance of a white, fluffy layer in the area where the chaplin peptide had been applied.

## Supporting Information

Figure S1 **FTIR microscopy of Δ** ***rdlAB*** **mutant** ***S. coelicolor*** **spores and aerial mycelium.** (A) Light microscopy image of aerial hyphae and spores of the *S. coelicolor* Δ*rdlAB* strain. (B) FTIR intensity in the amide I region for the same region; the coloured bar indicates high to low intensity (pink-blue respectively) and adjoining graphs represent the intensity of absorbance in the amide I region (1600 cm^−1^–1700 cm^−1^) in x- and y- dimensions in the position of the cross hairs (white dotted lines).
(TIFF)

Figure S2 **FTIR microscopy of Δ** ***chpABCDEFGH*** **mutant** ***S. coelicolor*** **spores and aerial mycelium.** (A) Light microscopy image of aerial hyphae and spores of the *S. coelicolor* Δ*chpABCDEFGH* strain. Hyphae can be clearly seen in the top right corner of the image. (B) FTIR intensity in the amide I region for the same region; the coloured bar indicates high to low intensity (orange-blue respectively) and adjoining graphs represent the intensity of absorbance in the amide I region (1600 cm^−1^–1700 cm^−1^) in x- and y- dimensions in the position of the cross hairs (white dotted lines). The intensity of the entire region is significantly lower than for either the wild type (Figure 5) or Δ*rdlAB* (Figure S1).
(TIFF)

Figure S3 **Observation of chaplin homodimers by ESI-TOF Mass Spectrometry.** (A) Mass spectrum showing ChpF [M+4H]^4+^ (m/z 1295.9) and [M+3H]^3+^ (m/z 1727.5) ions. Deconvolution revealed the presence of several species including ChpF monomers (approx. 40% abundance; mass 5181.33) and disulphide-bonded ChpF dimers (approx. 20% abundance; mass 10359.86, i.e. 2× monomer mass less 2H); other species could not be assigned. (B) Mass spectrum showing ChpH [M+5H]^5+^ (m/z 1024.7), [M+4H]^4+^ (m/z 1280.7) and [M+3H]^3+^ (m/z 1707.2) ions. Deconvolution revealed the presence of two species: ChpH monomers (approx. 90% abundance; mass 5119.95) and disulphide-bonded ChpH dimers (approx. 10% abundance; mass 10237.68, i.e. 2× monomer mass less 2H).
(TIFF)

Figure S4 **ThioflavinT fluorescence assay of chaplin fibril assembly.** The ThT assay reveals that fibril formation is rapid, proceeding with no discernable lag-phase. The rate of asembly is also concentration-dependent. Concentrations of the crude extract were 240 µg.ml^−1^ (black squares), 180 µg.ml^−1^ (open triangles) or 120 µg.ml^−1^ (black circles).
(TIFF)

Text S1 (DOC)
