# Supplementary figures and images for: The Assembly of Individual Chaplin Peptides from Streptomyces coelicolor into Functional Amyloid Fibrils
Source: PLoS One. 2011 Apr 19;6(4):e18839. doi: 10.1371/journal.pone.0018839 (PMC3079736; doi:10.1371/journal.pone.0018839)

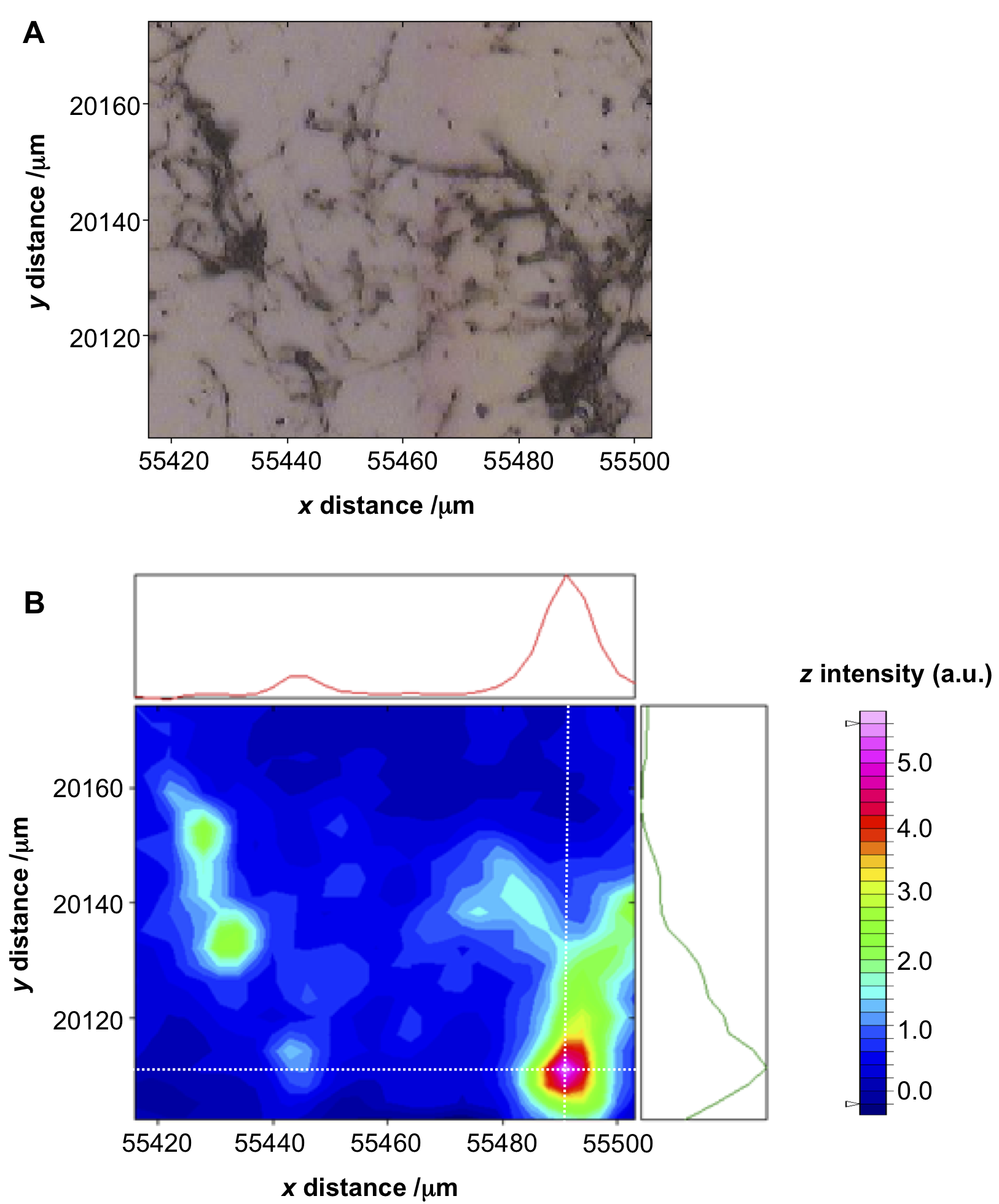

Supplement: Figure S1 — FTIR microscopy of Δ rdlAB mutant S. coelicolor spores and aerial mycelium. (A) Light microscopy image of aerial hyphae and spores of the S. coelicolor ΔrdlAB strain. (B) FTIR intensity in the amide I region for the same region; the coloured bar indicates high to low intensity (pink-blue respectively) and adjoining graphs represent the intensity of absorbance in the amide I region (1600 cm−1–1700 cm−1) in x- and y- dimensions in the position of the cross hairs (white dotted lines). (TIFF) [file pone.0018839.s001.tiff]

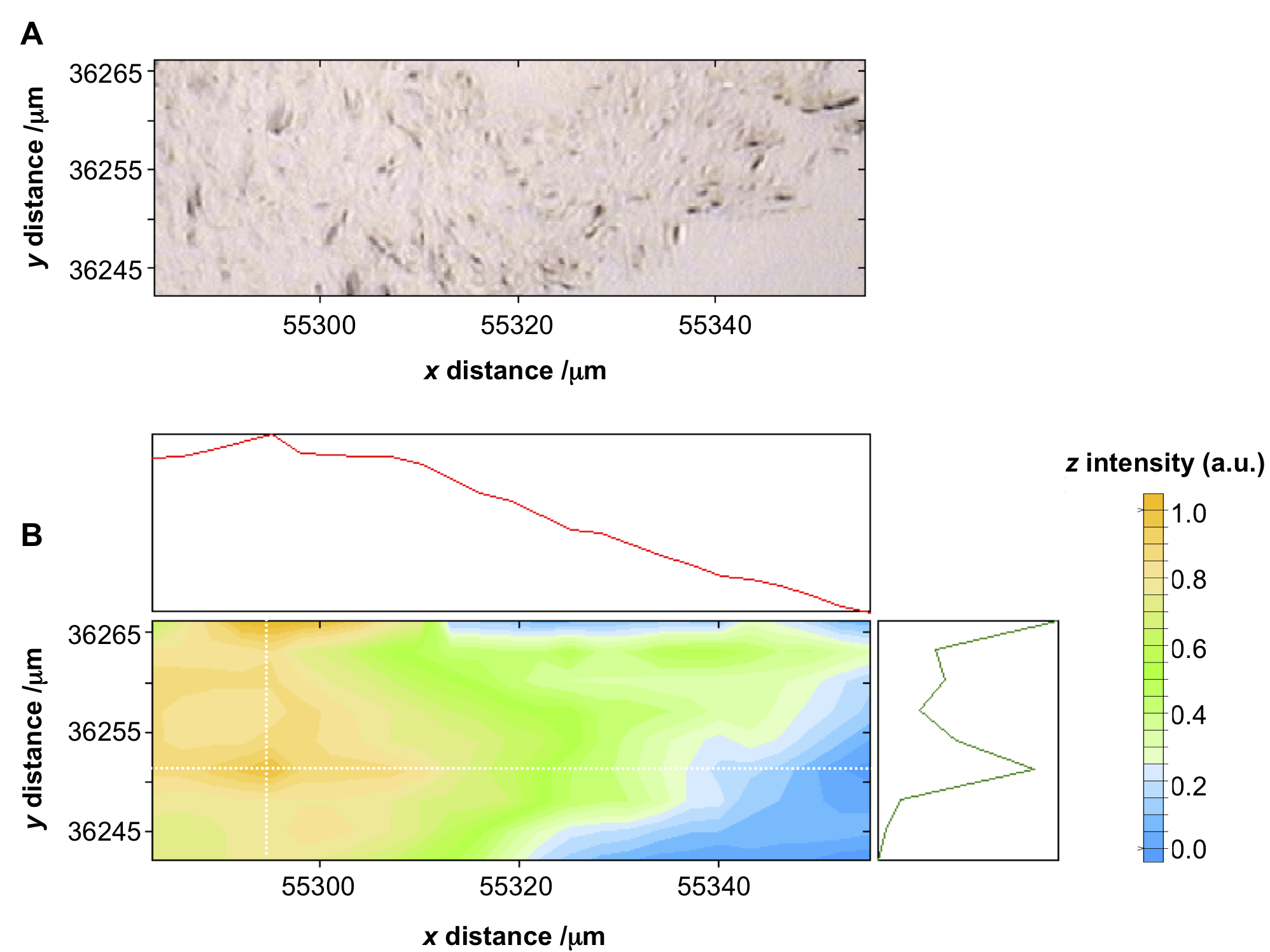

Supplement: Figure S2 — FTIR microscopy of Δ chpABCDEFGH mutant S. coelicolor spores and aerial mycelium. (A) Light microscopy image of aerial hyphae and spores of the S. coelicolor ΔchpABCDEFGH strain. Hyphae can be clearly seen in the top right corner of the image. (B) FTIR intensity in the amide I region for the same region; the coloured bar indicates high to low intensity (orange-blue respectively) and adjoining graphs represent the intensity of absorbance in the amide I region (1600 cm−1–1700 cm−1) in x- and y- dimensions in the position of the cross hairs (white dotted lines). The intensity of the entire region is significantly lower than for either the wild type (Figure 5) or ΔrdlAB (Figure S1). (TIFF) [file pone.0018839.s002.tiff]

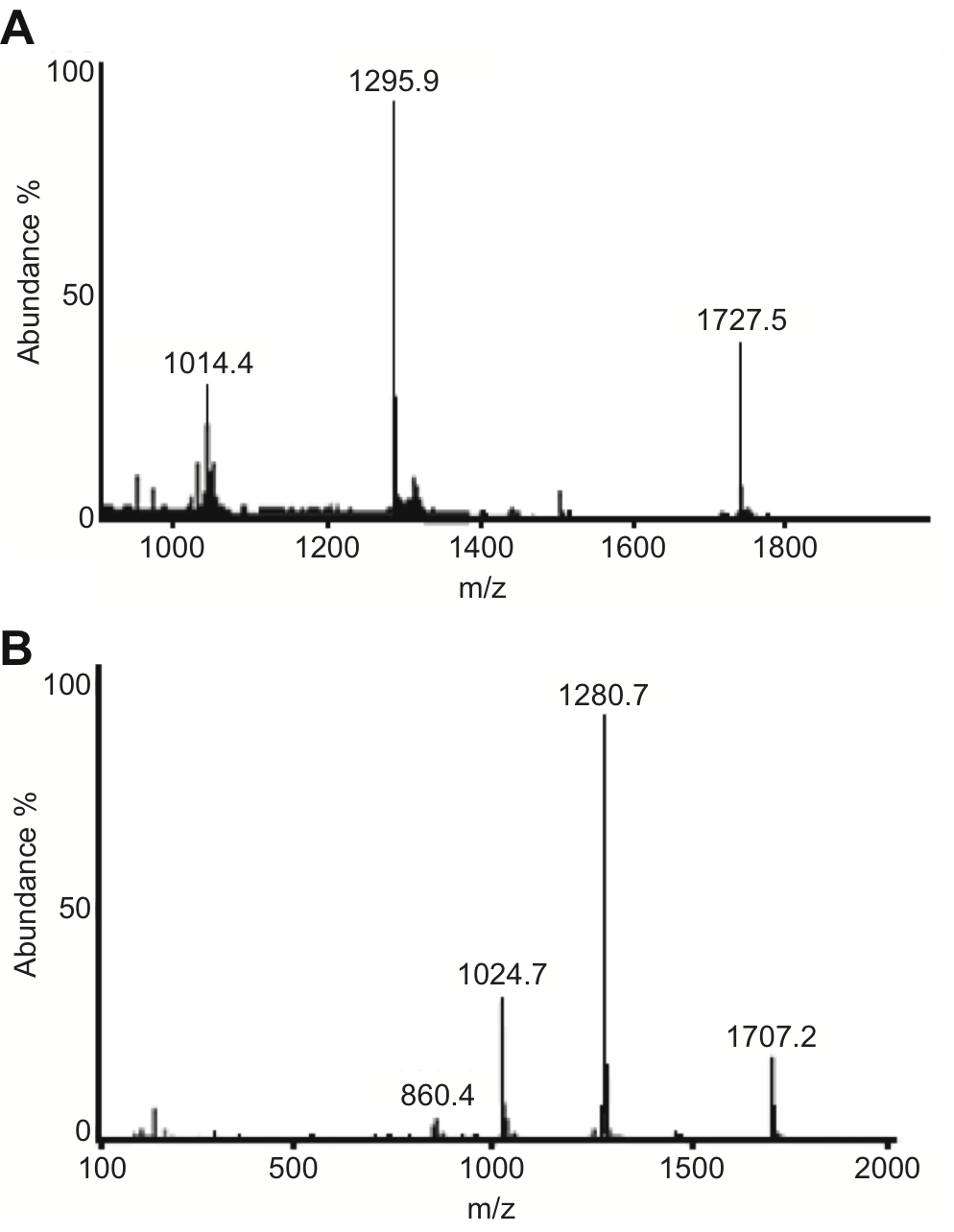

Supplement: Figure S3 — Observation of chaplin homodimers by ESI-TOF Mass Spectrometry. (A) Mass spectrum showing ChpF [M+4H]4+ (m/z 1295.9) and [M+3H]3+ (m/z 1727.5) ions. Deconvolution revealed the presence of several species including ChpF monomers (approx. 40% abundance; mass 5181.33) and disulphide-bonded ChpF dimers (approx. 20% abundance; mass 10359.86, i.e. 2× monomer mass less 2H); other species could not be assigned. (B) Mass spectrum showing ChpH [M+5H]5+ (m/z 1024.7), [M+4H]4+ (m/z 1280.7) and [M+3H]3+ (m/z 1707.2) ions. Deconvolution revealed the presence of two species: ChpH monomers (approx. 90% abundance; mass 5119.95) and disulphide-bonded ChpH dimers (approx. 10% abundance; mass 10237.68, i.e. 2× monomer mass less 2H). (TIFF) [file pone.0018839.s003.tiff]

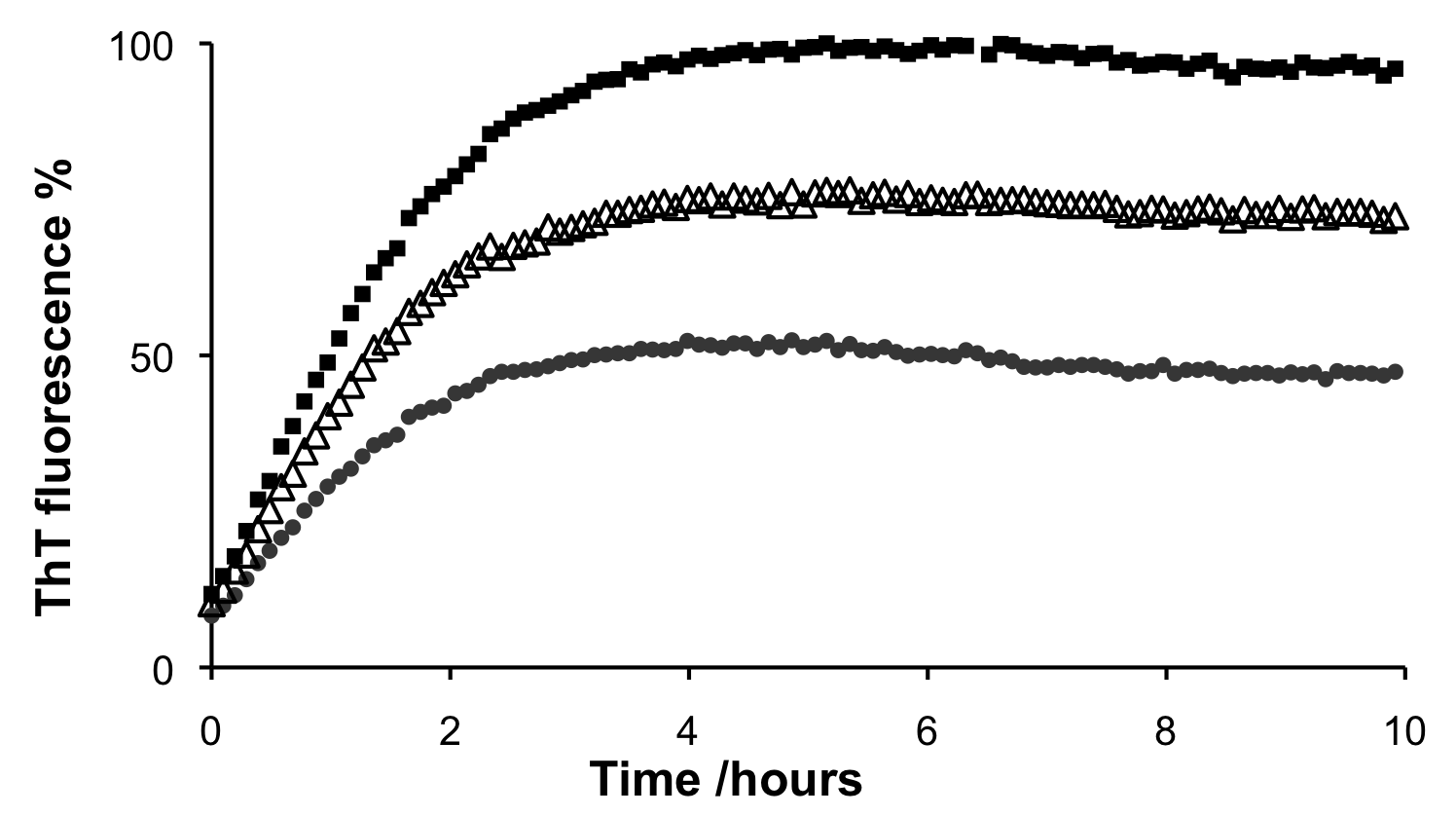

Supplement: Figure S4 — ThioflavinT fluorescence assay of chaplin fibril assembly. The ThT assay reveals that fibril formation is rapid, proceeding with no discernable lag-phase. The rate of asembly is also concentration-dependent. Concentrations of the crude extract were 240 µg.ml−1 (black squares), 180 µg.ml−1 (open triangles) or 120 µg.ml−1 (black circles). (TIFF) [file pone.0018839.s004.tiff]
