# Supplementary material for: The Assembly of Individual Chaplin Peptides from Streptomyces coelicolor into Functional Amyloid Fibrils
Source: PLoS One. 2011 Apr 19;6(4):e18839. doi: 10.1371/journal.pone.0018839 (PMC3079736; doi:10.1371/journal.pone.0018839)
Supplement: Text S1 — (DOC) [file pone.0018839.s005.doc]

## Supporting Information

## Materials and Methods

**Purification of Chaplins from *S. coelicolor* cell wall**

Chaplins were extracted from the cell wall using trifluoroacetic acid (TFA) as described by Claessen and co-workers [4]. The crude extract was dried under a gentle nitrogen stream, after which 500 μl of acetonitrile was added to the tubes to assist in the separation of peptide from any remaining cell wall. The preparation was centrifuged to remove any insoluble material and the supernatant was removed and freeze dried. The dry protein was then resuspended in the appropriate buffer for experiments requiring the whole extract. This extraction yielded 1 mg of protein for every 7 mg of cell wall, as assessed by the method of Bradford [35] using Bovine Serum Albumin as a standard. Individual chaplins were purified from the freeze dried mixture by high performance liquid chromatography (HPLC) using a Phenomenex Jupiter C18 column (250 x 14.6 mm) (Phenomenex, Torrance, CA, USA) and a linear gradient of water/acetonitrile with 0.1 % TFA with a flow rate of 1 ml.min^-1^. Absorbance was measured at 214 nm, 220 nm and 278 nm. Fractions were collected at an interval of 1 minute and MS confirmed the identities of the eluted peptides.

**Thioflavin T Assay**

A crude extract of proteins was prepared from the cell wall and the total protein quantified by Bradford assay [35] as described above. The extract was dissolved in 10 mM potassium phosphate, 150 mM NaCl, pH 7.0 at a concentration of 2 mg.ml^-1^ and different volumes of this solution were added to a black MicroWell 96-well plate (Nalge Nunc International, NY, USA) and diluted in the same buffer to give final crude extract concentrations of 240, 180 or 120 μg.ml^-1^. Thioflavin T was added to each well at a final concentration of 15 μg.ml^-1^. Spectra were recorded every 350 seconds using a FLUOstar Optima Microplate Reader (BMG LabTech, NC, USA) operating at 30 °C with orbital shaking at 100 rpm. Emission intensity at 480 nm was recorded; the extinction wavelength was 440 nm and bandwidths for excitation and emission were 10 nm. Data were normalised relative to the data series at highest protein concentration.
